# Stability of Liquid Films Formed by a Single Bubble and Droplet at Liquid/Gas and Liquid/Liquid Interfaces in Bovine Serum Albumin Solutions

**DOI:** 10.1021/acsomega.1c02188

**Published:** 2021-07-06

**Authors:** Dorota Gawel, Jan Zawala

**Affiliations:** Jerzy Haber Institute of Catalysis and Surface Chemistry Polish Academy of Sciences, ul. Niezapominajek 8, 30-239 Krakow, Poland

## Abstract

The properties of thin liquid films are usually investigated under static conditions, isolated from external disturbances. Such studies provide vital information about the drainage mechanism of the thin liquid film, but the conditions of these measurements are vastly different from those that occur when a real dispersed system is created. In this paper, we present elaborated methodologies that allow qualitative and quantitative measurements of the stability of both the emulsion and foam films formed by a single bubble and droplet at liquid/gas and liquid/liquid interfaces, where the hydrodynamic factors are of crucial importance. The experiments were performed in a bovine serum albumin (BSA) solution at different pH values. The adsorption behavior of BSA under different pH conditions at the liquid/gas and liquid/liquid interface is described, and its implication for the single bubble/droplet motion and liquid film drainage is analyzed. The mechanism of thin-liquid-film stabilization by the BSA molecules is shown to be significantly different for the foam and emulsion films and depends significantly on the bubble history as well as the pH of the BSA solution. Additionally, the results obtained for BSA were compared to those acquired for a typical surface-active substance, sodium lauryl sulfate. The similarities and differences in the rising bubble/droplet dynamics (caused by different dynamic adsorption layer architectures) and foam and emulsion film stabilization by these two types of stabilizers under dynamic conditions are shown and discussed.

## 1 Introduction

Emulsions and foams represent a group of dispersed systems widely encountered in industry, technology, separation processes, and everyday products. The common feature of these systems is their general structure: they are composed of thin liquid films, an elementary unit existing in every dispersed system with a liquid continuous phase.^1,2^ Investigating the properties of a single liquid film can be a useful probe to obtain significant information about the real system characteristics and stability.^3^

To date, the majority of studies on the properties of liquid films have been performed at a plane liquid/liquid interface under static conditions, where the liquid film was isolated from any external disturbances.^4−8^ Studies conducted under such a regime are extremely important as they allow for the determination of the drainage kinetics of the thin liquid film and the characterization of intermolecular forces. However, the aforementioned well-isolated static conditions induced in such experiments deviate from those that occur when foam or emulsion is created in real life. In the initial stage of the dispersed system, its internal conditions are highly dynamic. There are many motions, disturbances, and various dynamic processes that lead to bubble/droplet collisions, bouncing, and energy dissipation. Such dynamic conditions directly influence the drainage kinetics of the formed liquid films, which can be governed by motion-induced dynamic effects occurring at interacting interfaces (e.g., surface tension gradients). These dynamic effects act as “brakes” that slow down the process of liquid drainage to the critical thickness of rupture and thus prevent the process of coalescence of interacting droplets/bubbles.^9^ Under dynamic conditions, hydrodynamic factors such as dynamic adsorption layer and interface area changes determine the ability of thin liquid films to prevent initial disturbances and survive until static conditions are established, in which equilibrium thickness is reached and a liquid film can exist for a long period of time.^10−13^

It is well-known that to obtain a stable dispersed system, surface-active substances (surfactants) are required. The adsorption of surfactants at liquid/liquid or liquid/gas interfaces significantly changes the thin-liquid-film drainage kinetics, which is directly reflected in their stability and timescale of rupture. This is mainly the result of changes in the hydrodynamic boundary conditions. Under dynamic conditions, these changes can be pronounced, especially due to motion-induced surface tension gradients and Marangoni stresses.^9^ To stabilize the emulsion or foam films, various substances are used, including “classical” synthetic surfactants and their biodegradable counterparts such as proteins, polyelectrolytes, or lipid derivatives. Each of these groups of stabilizers has a different mechanism of adsorption and, consequently, stabilizes the thin liquid films in a different manner.^5^ In the present study, to compare these differences under dynamic conditions, sodium dodecyl sulfate (SDS) and bovine serum albumin (BSA) were chosen as models of well-characterized surface-active compounds. To control the degree of adsorption coverage and structure over the droplet/bubble surface by protein molecules, its pH-regulated adsorption activity and pH-dependent molecule conformation were exploited. The influence of pH on the BSA conformations in the bulk and at the liquid/liquid and liquid/gas interface has been extensively studied over the past few decades.^14−18^ It is generally accepted that in acidic and basic environments, molecules of BSA adopt expanded and asymmetric structures; in contrast, near the isoelectric point (IEP), their structure is rigid and compact.^19^ Furthermore, pH variations change the net charge of proteins, which can be either positive (under acidic conditions) or negative (under alkaline environments). Both factors determine the varying ability of BSA molecules to adsorb at the bubble/oil droplet interface.

This study investigates the influence of dynamic conditions on emulsion and foam-film stability. Accordingly, the impact of the state of the adsorption layer on the properties of the liquid film over the surface of the rising droplet/bubble was determined. The stability of the emulsion and foam films was analyzed using qualitative and quantitative methods, which reveal that the stabilization mechanism of BSA molecules is pH-dependent for both types of investigated thin liquid films formed under dynamic conditions. Additionally, a corresponding study was conducted for SDS as a typical surfactant. The similarities and differences between the properties of the liquid films stabilized by protein and SDS molecules under dynamic conditions are discussed in detail.

## 2 Materials and Methods

### 2.1 Materials

Dodecane (≥99%) was used as an oil (dispersed) phase, and SDS and BSA (M ∼66 kDa, >98% protein, essential fatty acid-free) were obtained from Sigma-Aldrich in the highest available purity. The pH of the BSA solution was adjusted by adding small amounts of high-purity NaOH and HCl (Sigma-Aldrich) at a concentration of 1 M. All reagents were used without further purification. The pH adjustment process was controlled using an Elmetron pH meter equipped with a standard glass electrode. BSA solutions were freshly prepared prior to each experiment. Four pH values—3.5 (acidic), 5 (close to the IEP of BSA molecules), 7 (native), and 9.5 (basic)—were chosen to investigate the impact of pH on the adsorption of BSA at the bubble and oil droplets and its implication for liquid stability under dynamic conditions. For interfacial tension measurements, the BSA solution concentration was kept constant at 7.5 × 10^–6^ M, similar to the rising droplet experiments. In the series of experiments with rising bubbles and the stability of foam films formed at the air/solution interface, the concentration of BSA was slightly lower (6 × 10^–7^ M). The solutions were prepared using ultrapure water (Milli-Q, 18 MΩ·cm). All parts of the experimental setup to be placed in contact with the prepared BSA solutions were thoroughly cleaned using a diluted solution of Mucasol Schulke (Sigma-Aldrich) cleaning liquid and rinsed with a large quantity of Milli-Q water. The measurements were performed at 21 ± 1 °C. The ionic strength of the solution varied according to the adjusted pH.

### 2.2 Experimental Setup

**Figure 1:**
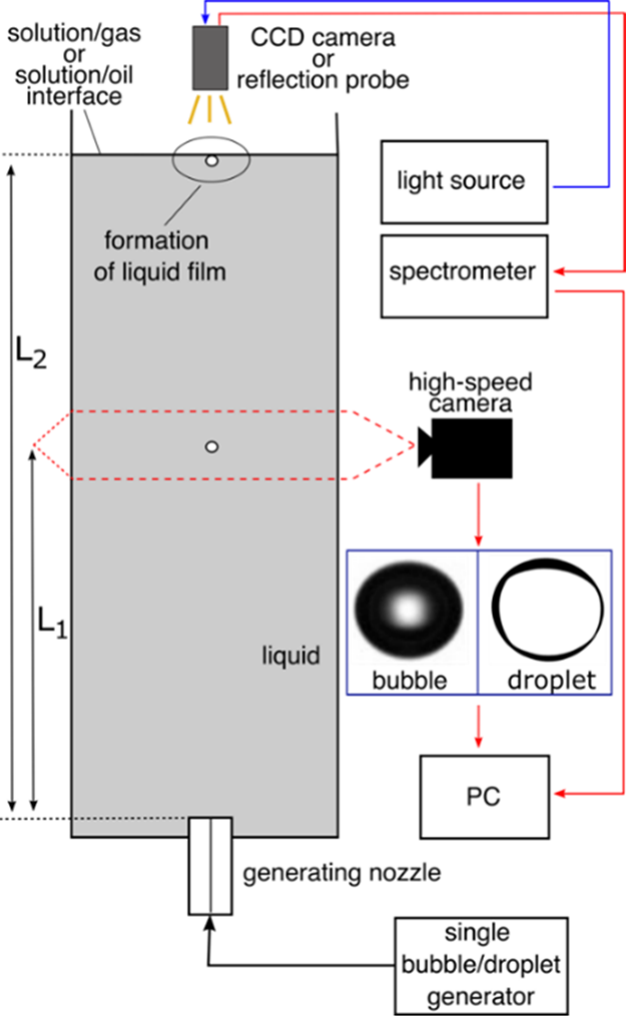
Illustration of the general experimental setup.

The general scheme of the experimental setup used to perform all experiments is presented in Figure 1. It consisted of three main parts: (i) a square glass column filled with the tested solution, with the generating nozzle sealed at the bottom; (ii) high-speed camera (SpeedCam Weinberger MacroVis) to monitor the single bubble/droplet motion (velocity); and (iii) a system for either qualitative or quantitative determination of the liquid-film (foam or emulsion) stability (kinetic of drainage) under dynamic conditions (i.e., at the early stage of its existence, after bubble/droplet collision with the interface). A single bubble or droplet was generated at the nozzle orifice using self-elaborated generators, which have been described in detail elsewhere.^20,21^ For air bubble generation, a thick-walled glass capillary of 0.15 mm inner diameter was used as the generating nozzle. The bubble diameter depended only on the solution surface tension and orifice diameter (1.9 mm), with only small deviations (less than 5%) related to the solution surface tension variations. In the case of the dodecane droplet, the three-way generating nozzle consisted of a glass tube and steel needle with an outer diameter of 0.51 mm, sealed concentrically.^21^A single droplet of adjustable size was formed at the needle tip owing to the adjusted overpressure in the glass cell filled with the oil phase. The final droplet size depended on the magnitude of the overpressure impulse from the oil cell. The overpressure impulse was kept constant in all experiments. Droplet detachment was forced by the short water flow impulse from the glass water cell, whose magnitude was controlled independently by the elaborated software. The moment of water impulse application (causing droplet detachment) could also be controlled, which allowed control of the droplet aging time, that is, the time available for surface-active substances to adsorb at the solution/oil interface (during the time of droplet residue at the needle tip).

The high-speed camera position was adjusted to approximately 17 cm (*L*_1_) above the bubble/droplet formation point (generating nozzle orifice). The acquired pictures of the rising bubble/droplet were used to calculate their velocities, which, at a distance equal to *L*_1_, were terminal (constant in time because of the establishment of steady-state conditions).

The single bubble/droplet reaching the solution interface, located ca. 25 cm (*L*_2_) above the generating nozzle, formed a liquid film. In the case of bubbles, a foam film was formed directly at the solution/air interface. To study the formation of an emulsion film, a thin layer (ca. 4 mm) of dodecane was spread on the solution surface.

For both foam and emulsion films, to focus the colliding bubble/droplet in the middle of the liquid column at the interface, a poly(tetrafluoroethylene) ring with an inner diameter of 27 mm was carefully immersed in the solution to form a convex meniscus at the solution/air or solution/oil interface. To assess the stability of the formed liquid films under dynamic conditions, two independent methods were applied: (i) determination of the lifetime of the droplet at the interface and (ii) direct measurements of the liquid-film drainage (i.e., determination of variations in the liquid-film thickness over time).

### 2.3 Bubble/Droplet Velocity Determination

**Figure 2:**
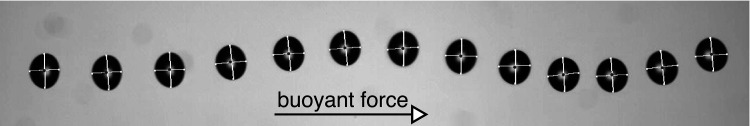
Example of rising velocity analysis (for bubble), taken during experiments.

Video monitoring of a single bubble/droplet rising in the tested solution allowed for the determination of variations in velocity. The values of the bubble/droplet velocity in the subsequent time steps (*u_i_*) were calculated using the following equation:
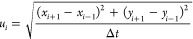
1where *x*_*i* + 1_, *x*_*i* – 1_, *y*_*i* + 1_, and *y*_*i* – 1_ are the coordinates of the momentary positions of the geometrical center of the bubble/droplet and Δ*t* is the corresponding time difference (calculated according to the camera frequency). As the distance covered by the bubble/droplet from its formation point is sufficiently long, the determined *u_i_* corresponded to the terminal velocity, and the final value was calculated as an average of all momentary *u_i_* values. The coordinates of the bubble/droplet geometrical center were calculated automatically using the self-elaborated Python script with the PIL module, allowing image analysis of the acquired pictures. An example of the analysis output for the rising bubble is shown in Figure 2. In addition to the *x* and *y* geometrical center coordinates, the vertical (*d_v_*) and horizontal (*d_h_*) diameters of the bubble/droplet were measured to calculate the equivalent diameter as follows:
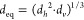
2

### 2.4 Qualitative Assessment of Emulsion Film Stability

**Figure 3:**
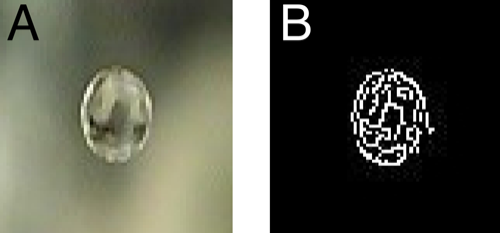
Single droplet beneath the solution/oil interface creating the emulsion film: (A) original image taken during our experiments and (B) image after contour extraction used in the lifetime calculation algorithm.

The liquid-film stability was qualitatively analyzed on the basis of the experimentally determined lifetime of a single droplet, that is, the time span between liquid-film formation (droplet collision with the interface) and its rupture (droplet coalescence), which was measured using a CCD camera mounted above the liquid column (solution/oil interface). The lifetime measurement was easily obtained: when the droplet appeared at the interface (i.e., when the liquid film was formed), elaborated software, based on the online image analysis performed using the Python OpenCV module, began to measure time. If the droplet ruptured and coalesced at the interface, information about its lifetime was automatically acquired. Figure 3 presents the original image of the droplet taken from above by the CCD camera (Figure 3A) and extracted contours (Figure 3B) used in the lifetime calculation algorithm. For reasonable statistics, the lifetime of a minimum of 50 single droplets was measured during one experimental run (i.e., one tested solution). The droplet lifetime could be associated with the drainage time of the emulsion film to the critical thickness of rupture, the point at which coalescence occurred between two liquid/liquid interfaces.

### 2.5 Direct Measurements of Average Liquid-Film Thickness

**Figure 4:**
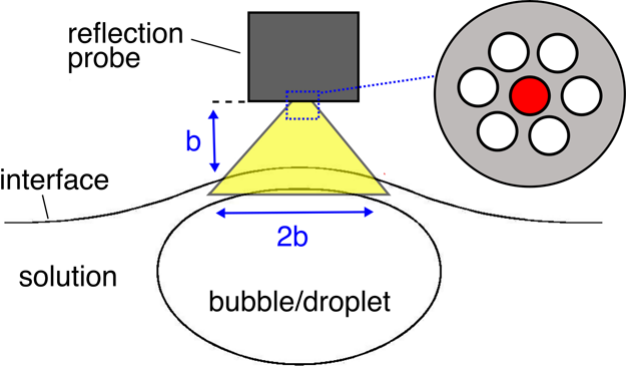
Schematic illustration of the liquid-film thickness measurements using an Ocean Optics reflection probe (five fiber optics transmit light to the sample and one in the middle receives interfering waves).

To quantitatively describe the kinetics of drainage of a single liquid film formed under dynamic conditions, an experimental protocol adapted from Delacotte et al.^22^ was applied. The measurements were performed using a reflection probe, which was connected to a light source operating in a wavelength range of 200–990 nm (Ocean Optics, DH-2000-BAL) and spectrometer (Ocean Optics, model QE Pro-ABS). For foam-film drainage determination, the probe was mounted just above the solution/air interface such that the location where the foam film was formed was illuminated (as schematically illustrated in Figure 4). In the case of emulsion films, the probe was immersed in the top layer of the oil phase. In both cases, the position of the probe above the bubble- or droplet-colliding spot (i.e., the location of liquid-film formation) was carefully adjusted to obtain a reasonable signal-to-noise ratio (which was extremely sensitive to even small deviations from the proper probe adjustment).

**Figure 5:**
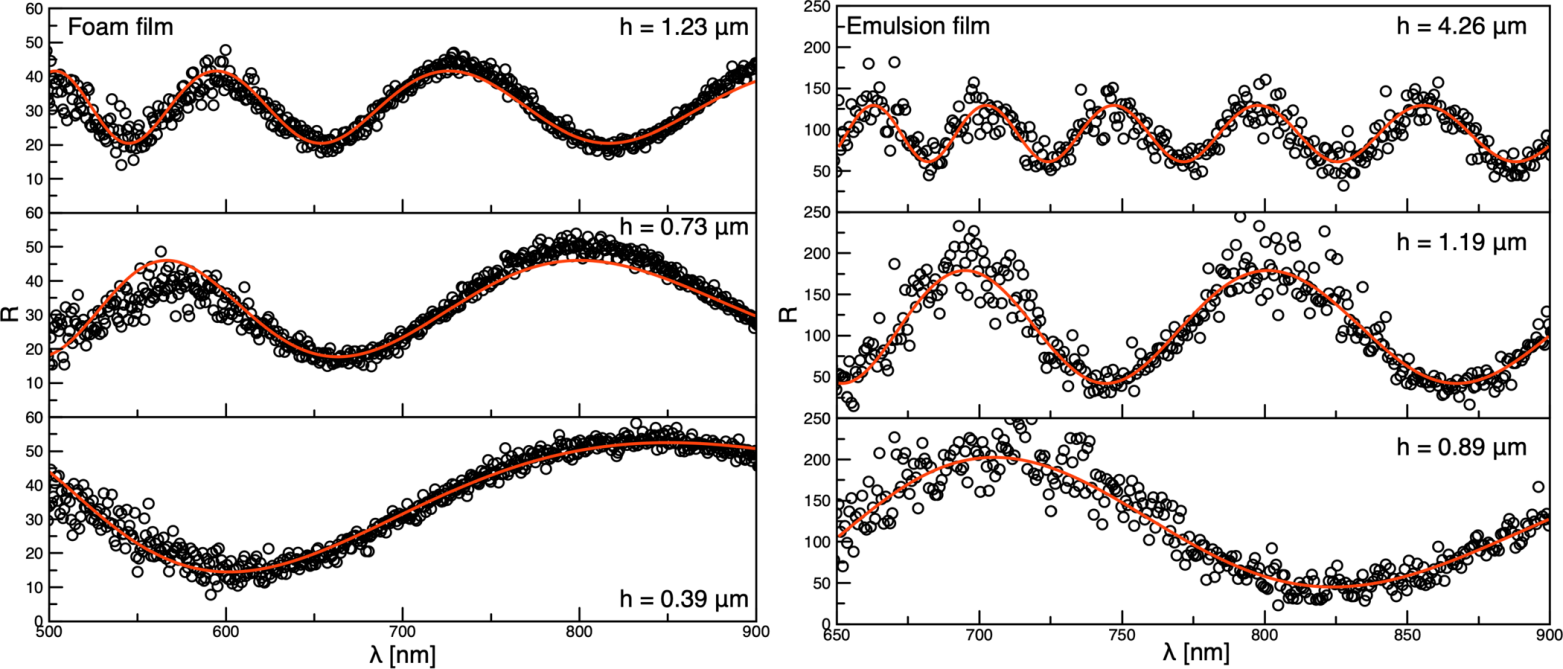
Examples of the experimentally obtained spectra (points) with the best fits (red lines) of eq 3. For each spectrum, the extracted value of the liquid-film thickness (*h*) is given.

The probe was designed in a way so as to simultaneously act as the light transmitter and light receiver (see Figure 4). Five outer fiber optics transmitted light to the sample from the light source, while the one in the center (marked in red in Figure 4) received the signal (interfering waves) transmitted to the spectrometer. Therefore, it was possible to acquire the interfering waves after their reflection from both interfaces, creating the liquid film in the form of sinusoidal spectra (see Figure 5: reflectivity *R* vs wavelength λ, recorded every 0.1 s). The recorded signals were then analyzed to determine the exact value of the liquid-film thickness, which involved fitting the following analytical equation^22^
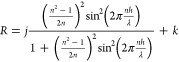
3to the chosen λ range of the experimentally obtained spectra, where *h* is the liquid-film thickness, *n* is the refractive index (1.33 and 1.39 for foam and emulsion films, respectively), and *j* and *k* are parameters of the fits. As one drainage curve was obtained by averaging the drainage kinetics of 20 individual liquid films and in every experimental run (for one liquid film) up to 500 spectra could be acquired (depending on the drainage kinetics), the analysis was automatized using a self-elaborated Python script with a nonlinear least squares fit approach (SciPy module). Figure 5 presents examples of the fits for the spectra obtained for the foam and emulsion films with an extracted value of *h*. As shown in Figure 4, it is noteworthy that the width of the detection window (the area from which the signal was acquired) depended on the distance between the probe and the interface. When the probe was adjusted to a distance *b* from the interface, the perimeter of the illuminated area was 2*b*. In practice, the position of the probe was adjusted to obtain the best noise-to-signal ratio; nevertheless, it can be safely assumed that the signal registered the entire liquid-film area. Therefore, the *h* values, which were determined from eq 3 and the fitting procedure, were averaged over the entire film radius.

### 2.6 Interfacial and Surface Tension Measurements

**Figure 6:**
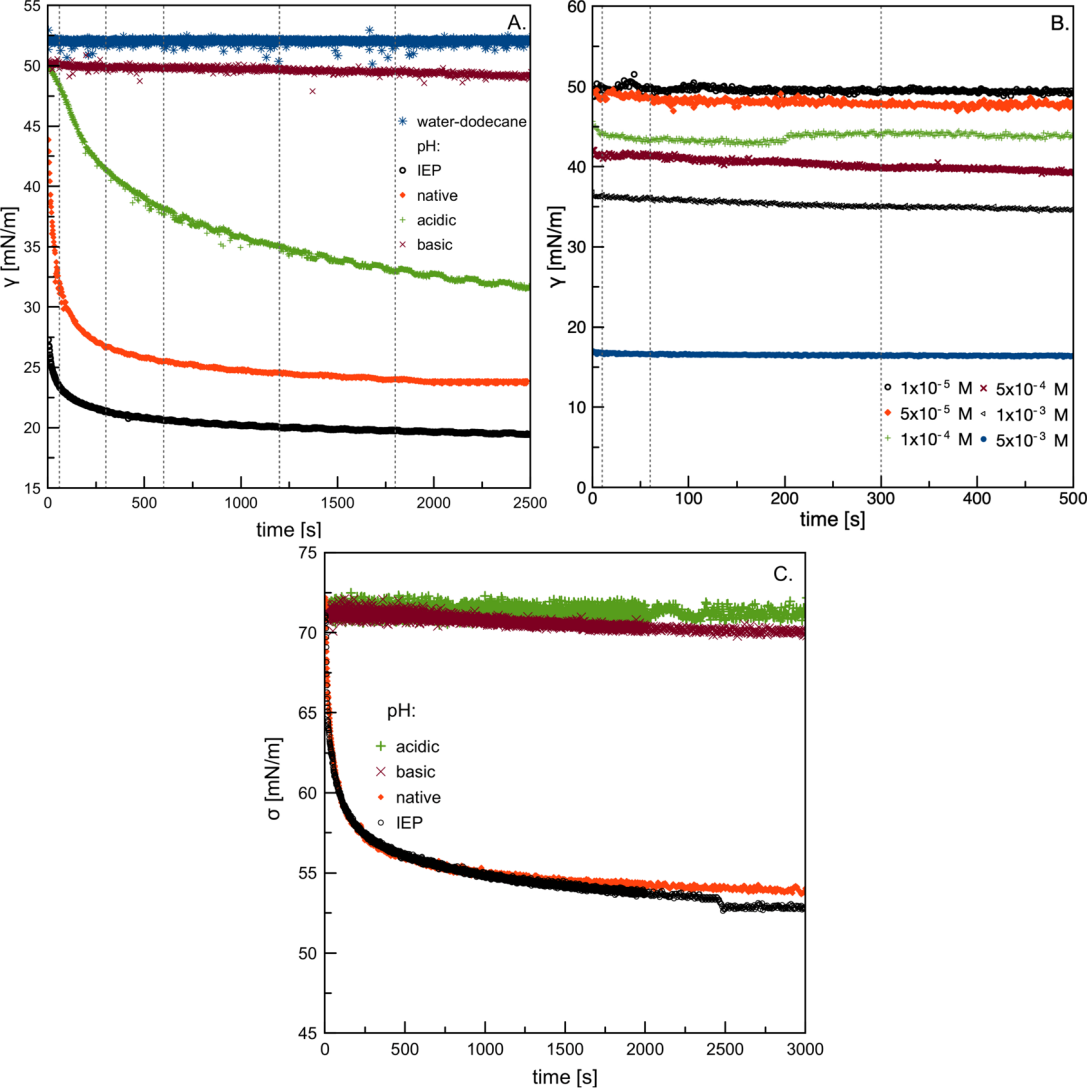
Interfacial tension of aqueous phase/dodecane interface measured for (A) 7.5 × 10^–6^ M BSA solutions at different pH values and (B) SDS solutions of different concentrations. (C) Evolution of the surface tension for solutions of 6 × 10^–7^ M BSA at various pH values.

Interfacial tension (IFT) in solutions having different concentrations of SDS and 7.5 × 10^–6^ M BSA at various pH values was measured using optical contact angle measurement and drop contour analysis 15EC (Data Physics, Germany) via the pendant drop method. The dodecane drop (≈20 μL) was formed using a syringe at the tip of a U-shaped stainless-steel needle immersed in a glass cuvette filled with the studied solution. The interfacial tension was calculated from the pendant droplet shape parameters acquired by the SCA software module using the Young–Laplace equation. In addition, the surface tension of 6 × 10^–7^ M BSA solutions was measured at various pH values by bubble profile analysis using PAT-1 (SINTERFACE Technologies, Germany). The variations in the measured IFT and surface tension values over time are shown in Figure 6; the average value of the interfacial tension between pure water and dodecane was determined to be 52 mN/m. It can be seen that for all concentrations of SDS, the adsorption equilibrium was attained quickly (up to ca. 300 s), and the IFT variations with time were relatively small, which is in good agreement with the literature data.^23,24^ In contrast, as expected, the BSA adsorption kinetics were much longer and depended on the pH conditions.

## 3 Results and Discussion

### 3.1 Comparison of Bubble/Droplet Rising Velocities

**Figure 7:**
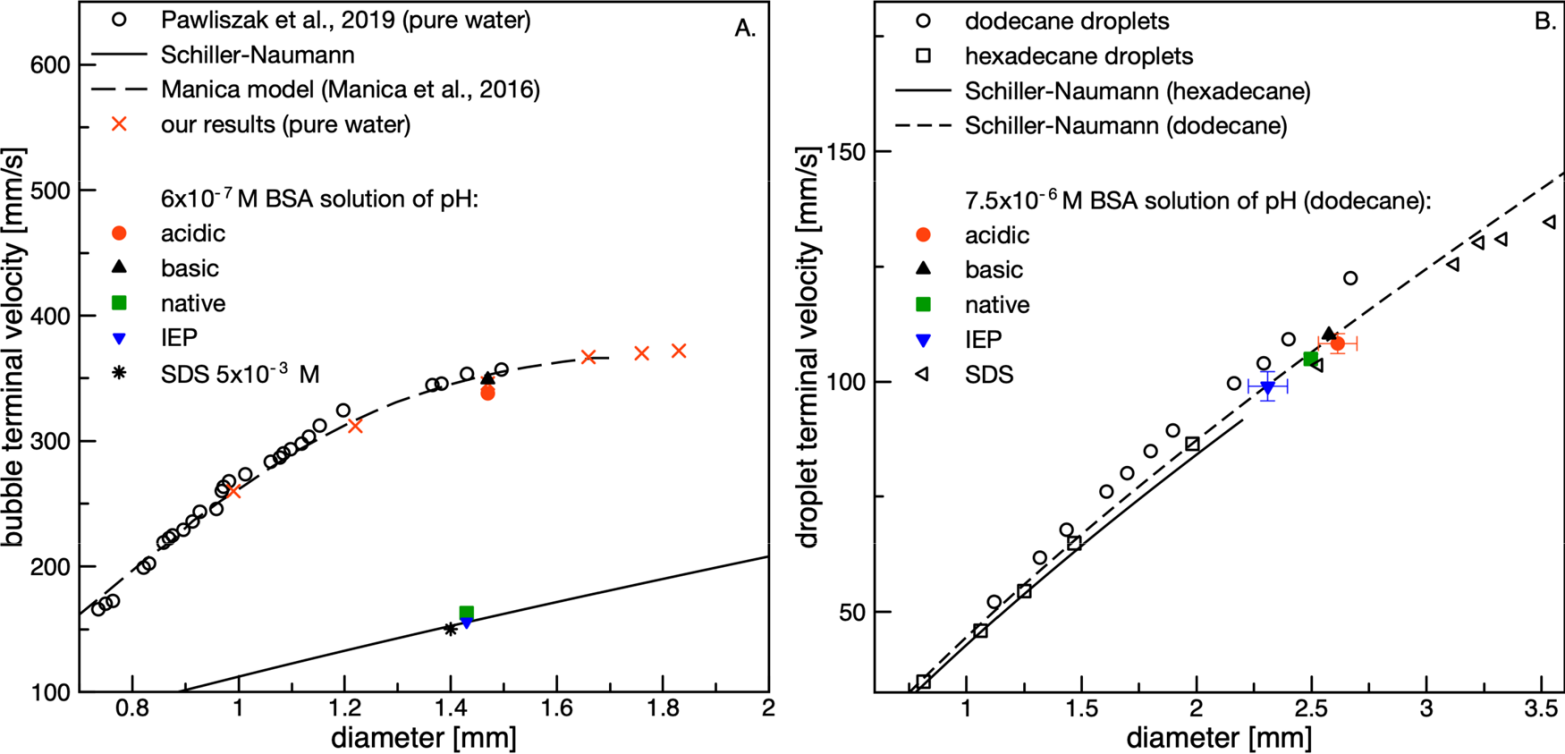
Variations in terminal velocity with an equivalent diameter of (A) bubble and (B) hexadecane and dodecane droplet (the dodecane droplet was used in experiments with all surface-active substances).

Figure 7 presents a comparison of the terminal velocities of bubbles and oil droplets rising in pure water, BSA solutions at different pH values, and SDS solutions of various concentrations. In the case of surface-active substances, the terminal velocities presented in Figure 7 were averaged for all the aging times studied during the experiments (the influence is discussed in a later section). In addition, we compared our experimental results with similar data obtained by Pawliszak et al.,^25^ a theoretical model developed by Manica et al.,^26,27^ and the classical Schiller–Naumann model.^28^ The model by Manica et al.^26,27^ can be used to describe bubble velocities for a wide range of diameters in a contamination-free system, that is, bubbles having fully mobile interfaces. The Schiller–Naumann model describes the velocity of a solid sphere of bubble size and density (no-slip conditions) and can be applied to a wide range of Reynolds numbers.

As seen in Figure 7, in the case of bubbles (Figure 7A), the experimentally obtained velocity values for pure water matched perfectly with the predictions of the model by Manica et al.^26,27^ and were consistent with the data presented in ref (25). This finding indicates that for the studied bubble size range, the hydrodynamic boundary conditions at the rising bubble surface could be considered as fully slip (i.e., the bubble interface was fully mobile). A similar situation was observed for BSA solutions of acidic and basic pH: BSA molecules were present in the solution but had no effect on the modification of the bubble surface mobility.

In turn, for the natural pH of the BSA solution and the pH close to the IEP (pH_IEP_), the measured bubble velocities were consistent with the Schiller–Naumann model, which indicated that the liquid/gas interface during bubble rise was completely immobilized (no-slip). The bubble behavior under these conditions was identical to that observed in the SDS solution with a relatively high concentration (5 × 10^–3^ M), which reveals that the liquid/gas fluidity retardation is caused by dynamic adsorption layer (DAL) formation.^10,29−31^

For the rising droplets (Figure 7B), the situation was different. In the case of pure water, up to ca. 1.5 mm droplet diameter, the dodecane and hexadecane droplets (shown here for comparison only) behaved like solid spheres (their velocities could be accurately described by the Schiller–Naumann model). However, above the threshold *d*_eq_ value, deviations from the Schiller–Naumann model predictions were observed. This very important observation indicates that for *d*_eq_ < 1.5 mm, the droplet surface was contaminated (there was a dynamic adsorption layer decreasing its fluidity), although pure water was used in the experiments. This is an important example of the “surface purity” issue raised by Pawliszak et al.,^25^ who showed that a real pure system does not exist, and the limit of detection of traces of impurities in water (no matter how thoroughly purified) depends on the rising bubble size. For bubbles with diameters <500 μm, the rising velocity can be predicted only by the Stokes^32^ or Schiller–Naumann^28^ formula (with a no-slip assumption). Agreement of the bubble velocity with the model assuming slip hydrodynamic boundary conditions was only observed for the bubble with *d*_eq_ > 0.8 mm. The situation presented in Figure 7B for oil droplets is analogous, but here, a much larger threshold diameter can be determined, most likely due to the potential presence of impurities in the continuous (bulk) and dispersed (oil) phases. The droplet surface can be considered as at least partially slip only for *d*_eq_ > 1.5 mm. Below this value, it is not possible to study the influence of any surface-active substance on the droplet motion parameters because the effects are negligible. This was the main reason we studied relatively large droplets (*d*_eq_ > 2 mm) in our experiments with surface-active substances.

The analysis presented above also explains the different influence of the BSA solution pH on the dodecane droplet velocity compared to bubbles. For droplets, independent of the solution pH, the presence of BSA immobilized the solution/dodecane interface completely (by inducing the no-slip hydrodynamic boundary conditions), similar to varying the SDS concentration from 1 × 10^–5^ to 5 × 10^–3^ M (the details of the SDS solution concentration and dodecane droplet velocities are given in Table 1). The dissimilarity of pH-dependent hydrodynamic boundary conditions at the solution/air and solution/dodecane interface is most likely a consequence of a much smaller gap between droplet velocities in water (where a slip or at least partial-slip boundary conditions can be assumed) and velocities for the fully immobilized solution/oil interface (Schiller–Naumann model). For dodecane droplet velocities measured in SDS solutions of various concentrations, small deviations from the Schiller–Naumann model can be observed. This is most likely a consequence of both the deviation from rectilinear droplet motion during the experiments and the dissolution of dodecanol (non-ionic contaminant from the SDS hydrolysis) in dodecane, which changes the physicochemical properties of the oil.^24^

**Table 1:** Equivalent Diameter and Terminal Velocities of a Droplet Rising in SDS Solutions of Various Concentrations

*c* [M]	*d*_eq_ [mm]	terminal velocity [mm/s]
1 × 10^–5^	3.54 ± 0.01	134.7 ± 0.6
5 × 10^–5^	3.33 ± 0.01	131.0 ± 0.7
1 × 10^–4^	3.12 ± 0.01	125.5 ± 0.4
5 × 10^–4^	3.23 ± 0.01	130.2 ± 0.9
5 × 10^–3^	2.53 ± 0.01	103.6 ± 0.4

**Figure 8:**
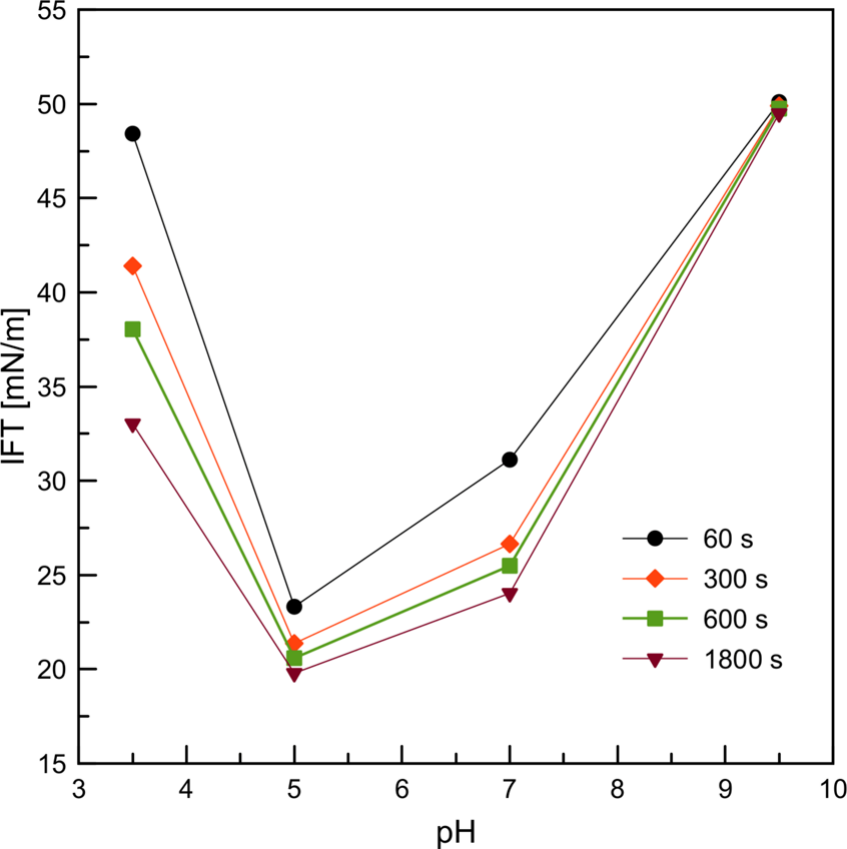
Dodecane/BSA solution interfacial tension as a function of pH for different droplet aging times.

The adsorption performance of the BSA molecules under different pH conditions is presented in Figure 8, and the IFT values for different droplet aging times are reported in this section. Independent of the chosen aging time, a similar situation occurs: the ability of BSA to decrease the dodecane/solution IFT was strongest for pH_IEP_ (pH = 5) and native pH = 7. For acidic pH (3.5), the IFT decrease was noticeable. In addition, the strongest influence of the aging time on the IFT values was observed. For basic pH (pH = 9.5), the IFT was constant and practically equal to the IFT of the dodecane/water interface, which indicates that the adsorption capability of the BSA molecules at this pH is totally hindered.

**Figure 9:**
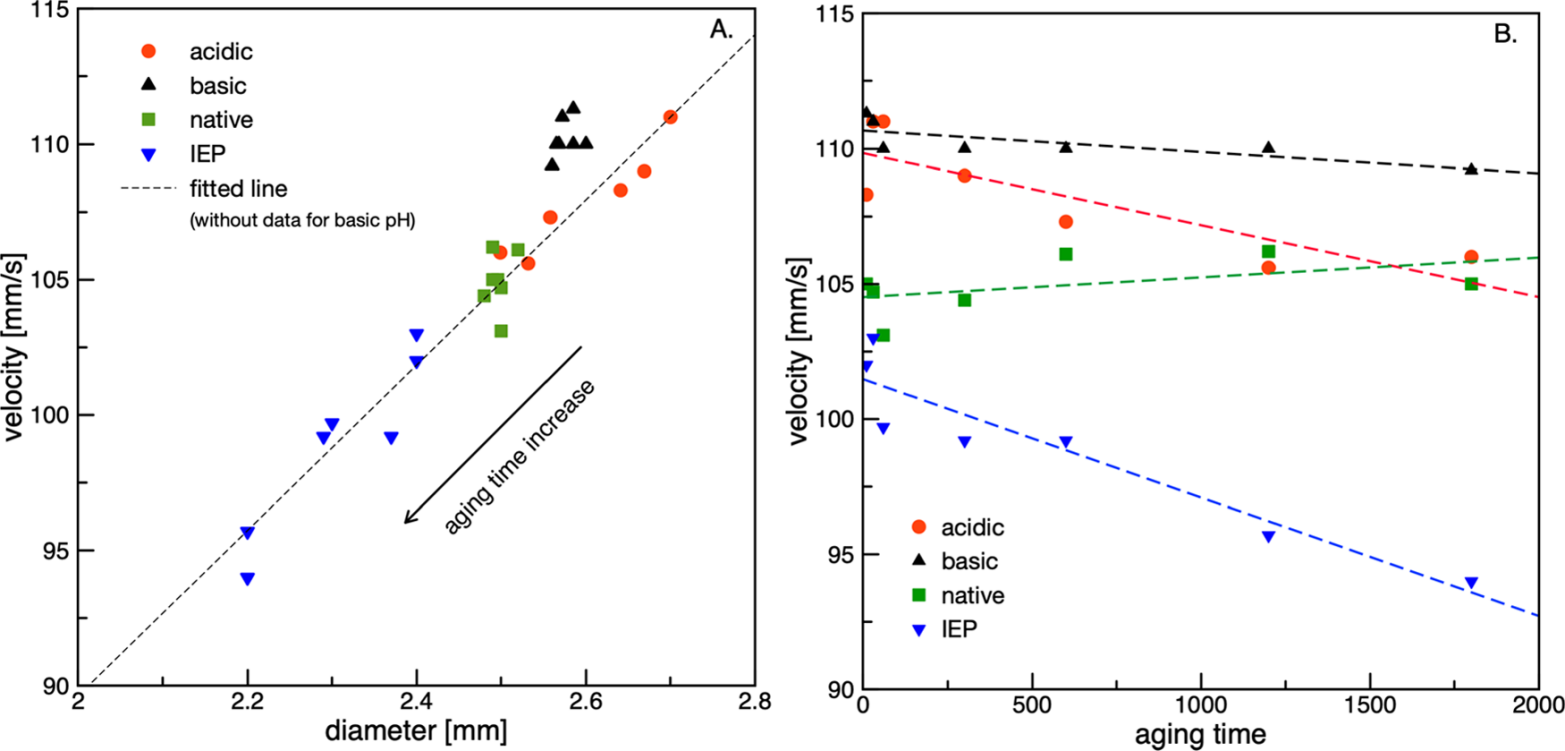
Influence of aging time on (A) droplet diameter and (B) velocity for rising oil droplets in BSA solutions of varying pH.

Figure 9 presents a more detailed analysis of the influence of the BSA adsorption capability on the droplet diameter and velocities. The points represent the experimental data, while the dashed lines indicate the fitted linear regression. In Figure 9A, the line was fitted to all points, except for those of the basic pH. As shown, various aging times are associated with different diameters of the droplets detached from the needle tip. This was due to keeping the pressure impulse constant during droplet generation for all experimental runs rather than correcting it for variations in the interfacial tension (see Figure 6). Constant pressure in the generating system implies that during the aging time, droplet shrinkage occurs, which is proportional to the ongoing adsorption and related changes in the interfacial tension values. The differences in droplet diameters are clearly relatively small, but it can be presumed that this phenomenon is probably the result of building adsorption layers of different structures at the oil droplet for each of the studied pH values. The strongest effect on the droplet diameter was observed for acidic pH and pH_IEP_. For these pH conditions, the broadest range of diameters, and consequently, velocities, was also observed. Surprisingly, for natural (native) BSA solution (pH = 7), the diameter and velocity barely changed with aging time. The same result was found for basic pH conditions.

This result was unexpected, considering the similar interfacial tension dependence for native and pH_IEP_ conditions (see Figure 6A). For pH_IEP_, the strongest effect of aging time on the diameter and velocity correlates well with the BSA adsorption behavior and the rate of the interfacial tension decrease.

**Figure 10:**
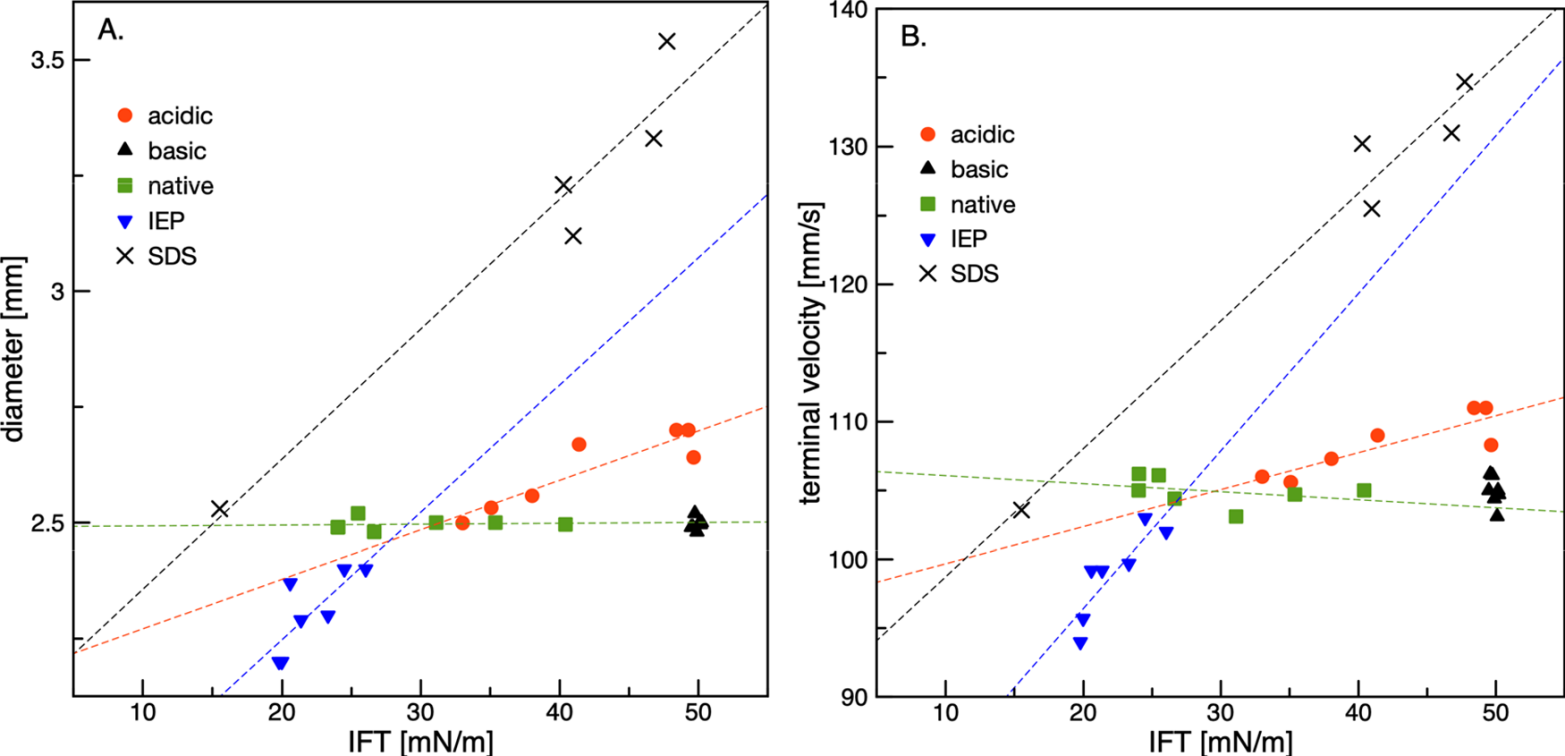
Rising droplet (A) diameter and (B) terminal velocity as a function of interfacial tension values corresponding to the aging times.

Aging times of 10, 30, 60, 300, 600, 1200, and 1800 s were selected for the experiments. Owing to the different timescales of the interfacial tension variations for the BSA and SDS solutions (shown in Figure 6), the chosen aging time range was tested in the experiments with BSA, while the time range for SDS was only 10–300 s. Figure 10 illustrates the influence of interfacial tension on the diameter and terminal droplet velocity. The IFT values presented on the *x*-axis correspond to the aging time values and were taken directly from Figure 6 (see the vertical dashed lines marked here). The lines in Figure 9 are linear regressions fitted to the experimental data; the determined ranges of the diameter variations correlate well with the IFT values. The analysis revealed that the best adsorption performance of the BSA molecules, leading to a significant decrease in the diameter of the formed droplets and a decrease in the droplet rising velocity, can be expected at pH_IEP_, which is comparable to that of a typical surfactant (e.g., SDS). Figure 10 also confirms that practically no adsorption of the BSA molecules occurs at the growing and rising droplets under basic pH conditions. Almost no variation in the IFT caused practically constant diameter and velocity. However, this velocity differed from that in water, indicating full immobilization of the rising droplet surface.

This difference was most likely caused by the surface-active impurities in the BSA sample, which exerted an effect when the adsorption activity of the BSA molecules was suppressed by the high pH value. Surprisingly, despite a rather broad range of IFT variations for the natural pH, no variations in diameter, and consequently, velocity, were observed, which confirms the results presented in Figure 8. To understand this unexpected trend, the differences in the formed structure of the adsorption layer over the oil droplet under acidic, native, and pH_IEP_ conditions during aging should be analyzed in more detail. It is worth highlighting that the influence of aging time on the properties of emulsion films stabilized by BSA was investigated in the previous literature;^4,6,33^ however, the importance of aging time in the movement of oil droplets has never been reported.

The presented results show that different droplet velocities were a consequence of the decrease in droplet diameter caused by the IFT decrease. No rheological anomalies (besides the unexpected trends revealed for natural pH), which can result from the viscoelastic network or bilayer structure formation at the solution/oil interface, appeared during testing, and BSA behaved similar to a typical surfactant, whose surface activity is pH-dependent and can be tuned by varying the pH. In addition, it was shown that despite the negligible affinity of the BSA molecules to adsorb to the solution/oil interface at basic pH values, the rising droplet surface can be completely immobilized, most likely due to impurities present in the sample.

### 3.2 Liquid-Film Drainage

**Figure 11:**
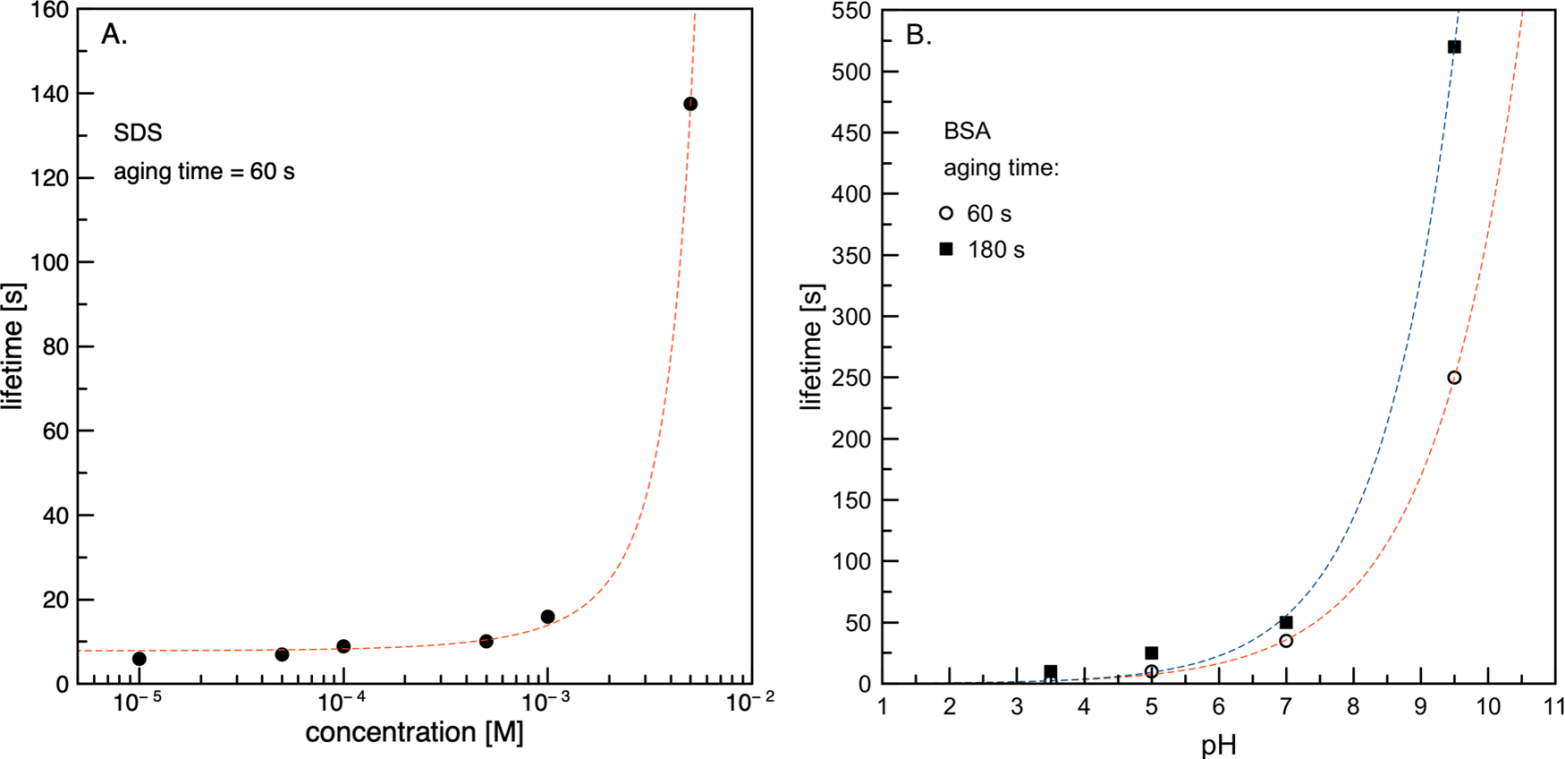
Comparison of obtained lifetimes of a single oil droplet in (A) SDS and (B) BSA solutions.

Figure 11 depicts the lifetimes of a single droplet at the solution/oil interface, which can be directly associated with the drainage kinetics of an emulsion film formed by a colliding droplet. For SDS, an aging time of 60 s was chosen, while for a BSA solution with a concentration of 7.5 × 10^–6^ M, the chosen time was 60 and 180 s. The relative standard deviation (RSD) of the average points shown in Figure 11 varied between 10 and 25%. In general, there are no noticeable differences in the presented trends for both substances, except that in the case of SDS, the gradual lifetime increase was caused by concentration variations (for BSA, pH changes were the cause). This increase was initially quite small and rose significantly for the highest SDS concentration and for the BSA solution with pH = pH_IEP_. Moreover, for an aging time of 60 s, the lifetime values for 5 × 10^–3^ M SDS and BSA solutions of pH = pH_IEP_ were of similar magnitude (ca. 140 and 230 s, respectively). For an aging time of 180 s, the measured lifetime was more than two-fold higher, revealing significant changes in the emulsion film drainage kinetics.

**Figure 12:**
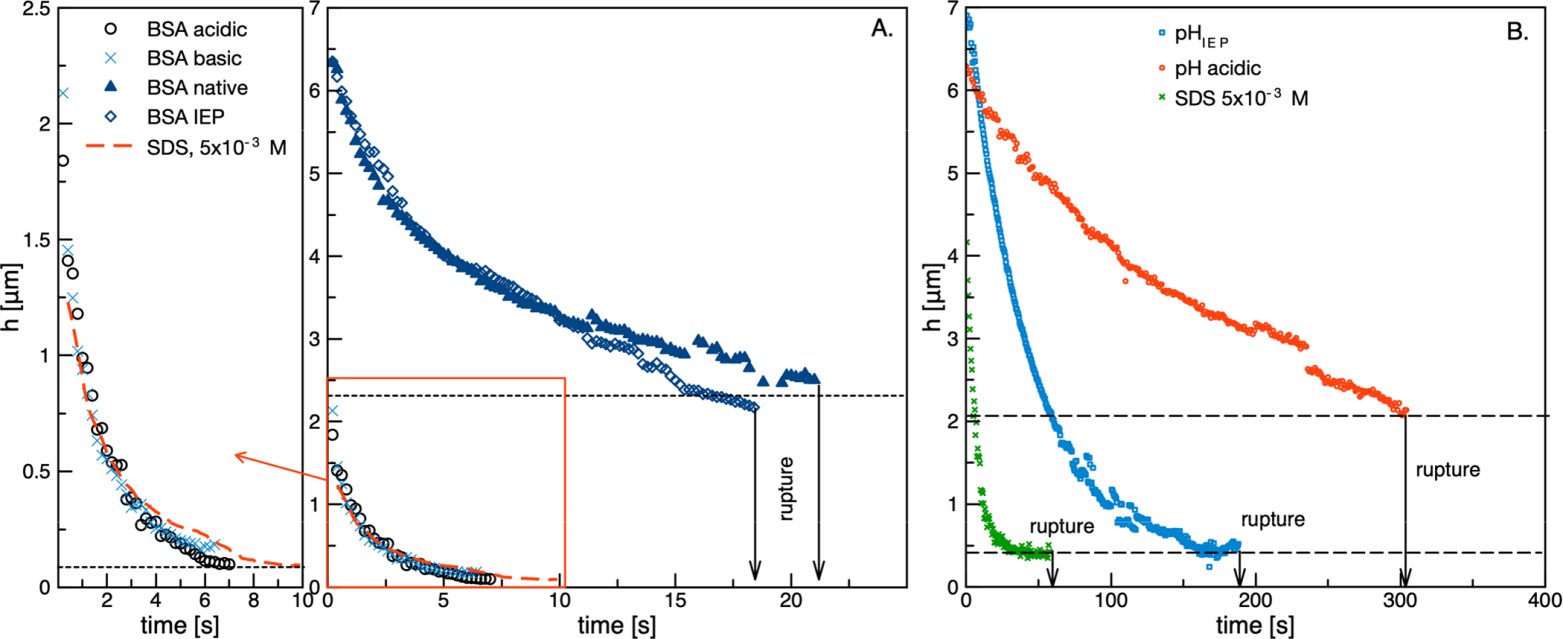
Drainage curves (thickness of liquid film vs time) of (A) foam and (B) emulsion films.

Figure 12 presents the data corresponding to those presented in Figure 11 but obtained using the interferometric method and protocol described in the Materials and Methods. This allowed for the direct determination of the liquid-film thickness formed under dynamic conditions (quantitative description). In addition, the drainage curves of a single foam film formed under dynamic conditions are presented. In the case of foam films, it can be seen that despite the identical ability of 5 × 10^–3^ M SDS to modify the rising bubble hydrodynamic boundary conditions, its efficiency in stabilizing foam films is significantly lower compared to the BSA solution of native pH and pH close to the IEP value. This result is comparable with those for BSA under acidic and basic pH levels, where slip boundary conditions were assumed at the rising bubble surface. This clearly indicates that the structure of the dynamic adsorption layer formed at the rising bubble surface was sufficient for the full immobilization of the liquid/gas interface during the bubble motion period (when the Reynolds number was on the order of 200). However, after liquid-film formation, the hydrodynamic boundary conditions at the top interface forming the liquid film were significantly shifted toward more slip. This finding is consistent with previous studies^34−36^ where a clear correlation between the liquid-film stability and the distance covered by the bubble before the formation of the foam film was reported. These studies showed that owing to the formation of a dynamic adsorption layer at the rising bubble surface (under steady-state conditions), the top bubble pole was practically devoid of surfactant molecules (depletion zone). Therefore, the top interface forming the liquid film has significantly lower adsorption coverage, and its fluidity has been retarded to a significantly smaller degree during liquid-film drainage.

The close correlation of the foam-film drainage curve determined for 5 × 10^–3^ M SDS with those obtained for basic and acidic BSA solutions indicates a similar mechanism, except that in this case, the main impurity present in the sample was the foam-film stabilizer. For BSA solutions of native pH and pH = pH_IEP_, much slower drainage indicates that the dynamic adsorption layer structure at the bubble surface rising in the BSA solution is completely different than expected for typical surfactants. The rheological phenomena, having no significance for the modification of the bubble rising velocities, are of crucial importance for foam-film drainage kinetics. This effect, arising from completely different DAL architecture in solutions of proteins (not yet reported in the literature), is certainly worth further investigation and is currently underway using the methods described in this paper.

In the case of emulsion films, it can be seen in Figure 12B that the slowest drainage occurred for the acidic pH. The film rupture at high thicknesses was approximately 2 μm. For pH_IEP_, the emulsion film drained faster and reached an equilibrium thickness of 430 nm, similar to the thickness determined for 5 × 10^–3^ M SDS solution. This value is larger than the rupture thickness determined for foam films in SDS solution and in acidic and basic BSA solutions (where the critical thickness was equal to ca. 100 nm). However, this equilibrium thickness is consistent with that of foam films reported by Lin et al.^37^ in aqueous BSA solutions, in which drainage was measured between a bubble and a bulk air/solution interface. The authors reported an equilibrium thickness on the order of 500–600 nm, with minimum and maximum values (due to dimple formation) of approximately 400 and 900 nm, respectively. In our case, this relatively high thickness is most likely a consequence of large liquid-film nonhomogeneities, which, according to the literature,^3,37−42^ can be highly significant in protein-stabilized systems. Under dynamic conditions, even higher thickness fluctuations are expected. The high rupture thickness of the emulsion film formed under acidic pH conditions suggests that the thickness nonhomogeneities were significantly higher compared to when pH = pH_IEP_.

It is noteworthy that the data presented in Figure 12A correlate perfectly with the foamability experiments presented in ref (14). The data show that independent of the BSA solution concentration, the foam height was the largest for pH levels close to the IEP value of the BSA molecule, and practically no foam was observed for acidic and basic pH levels. The correlation between the stability of a single liquid film and the real system is not a new observation and has been reported in many previous studies.^4,14,33,43^ However, we believe that the protocol presented here can also be successfully used to assess the stability of real-life foam and emulsion systems, especially at their initial stage of formation and existence, where conditions are dynamic and important hydrodynamic factors (such as the formation of a dynamic adsorption layer) are crucial for dispersed system formation probability.

## 4 Conclusions

The present work demonstrates the significance of dynamic conditions for the properties of thin-emulsion and foam films and their stability in the presence of proteins and surfactants. It was shown that both rising velocities and drainage kinetics are extremely useful probes for the differences in hydrodynamic boundary conditions at liquid/gas and liquid/liquid interfaces, allowing the comparison of the adsorption behavior of surfactants and proteins.

The experiments revealed that the ability to decrease the rising droplet terminal velocities in BSA solutions of pH = pH_IEP_ and natural pH is comparable to that of a typical surfactant (e.g., SDS). However, the mechanism of interface immobilization can be completely different. The foam-film drainage kinetics (both qualitative and quantitative) suggest that for SDS solutions, the bubble surface was immobilized as a result of the so-called DAL formation, which was similar for acid and basic BSA solutions. Significantly slower drainage for acidic pH and pH = pH_IEP_ suggests a completely different architecture of the adsorption layer at the moment of liquid-film formation. Therefore, the classical mechanism of interface immobilization most likely does not hold for a solution of proteins with high adsorption performance. This is an interesting result worthy of further investigation, including conducting experiments on bubble/droplet lifetime in protein solutions of various concentrations and pH levels and comparing the results to typical ionic and non-ionic surfactants.

Analysis of the velocity of droplets rising in the basic BSA solution indicated that despite the negligible adsorption performance of the protein molecules at the dodecane droplet interface, the studied solution/oil hydrodynamic boundary conditions were fully no-slip, which evidences the significant influence of impurities. This is an important, yet very often overlooked fact to keep in mind, especially for experiments under dynamic conditions, where even minor traces of surface-active substances can have huge hydrodynamic consequences. The quantitative investigation of liquid films formed under dynamic conditions in solutions of proteins revealed high inhomogeneity regardless of pH. Films created under these conditions had an average rupture thickness much larger than that typically observed for common synthetic surfactants. Moreover, the differences in the drainage kinetics of foam films under different pH conditions of the BSA solution correlate almost perfectly with the results of foamability tests reported in the literature. This verifies the effectiveness of our elaborated protocols for assessing the stability of real-life systems and their probability of formation.
